# Fetal cardiac function at intrauterine transfusion assessed by automated analysis of color tissue Doppler recordings

**DOI:** 10.1186/s12947-020-00214-1

**Published:** 2020-08-13

**Authors:** Lotta Herling, Jonas Johnson, Kjerstin Ferm-Widlund, Fredrik Bergholm, Peter Lindgren, Sven-Erik Sonesson, Ganesh Acharya, Magnus Westgren

**Affiliations:** 1 Department of Clinical Science, Intervention and Technology - CLINTEC, Karolinska Institutet, Stockholm, Sweden; 2 Center for Fetal Medicine, Department of Obstetrics and Gynecology, Karolinska University Hospital, Stockholm, Sweden; 3 Department of Medical Engineering, School of Technology and Health, KTH Royal Institute of Technology, Stockholm, Sweden; 4 Pediatric Cardiology Unit, Department of Women’s and Children’s Health, Karolinska University Hospital, Stockholm, Sweden; 5 Women’s Health and Perinatology Research Group, Department of Clinical Medicine, UiT-The Arctic University of Norway, Tromsø, Norway

**Keywords:** Fetal cardiac function, Tissue Doppler imaging, Automated analysis, Intrauterine transfusion, Fetal anemia

## Abstract

**Background:**

Fetal anemia is associated with a hyperdynamic circulation and cardiac remodeling. Rapid intrauterine transfusion (IUT) of blood with high hematocrit and viscosity into the umbilical vein used to treat this condition can temporarily further affect fetal heart function. The aim of this study was to evaluate the short-term changes in fetal myocardial function caused by IUT using automated analysis of cine-loops of the fetal heart obtained by color tissue Doppler imaging (cTDI).

**Methods:**

Fetal echocardiography was performed before and after IUT. cTDI recordings were obtained in a four-chamber view and regions of interest were placed at the atrioventricular plane in the left ventricular (LV), right ventricular (RV) and septal walls. Myocardial velocities were analyzed by an automated analysis software to obtain peak myocardial velocities during atrial contraction (Am), ventricular ejection (Sm), rapid ventricular filling (Em) and Em/Am ratio was calculated.

Myocardial velocities were converted to z-scores using published reference ranges. Delta z-scores (after minus before IUT) were calculated. Correlations were assessed between variables and hemoglobin before IUT.

**Results:**

Thirty-two fetuses underwent 70 IUTs. Fourteen were first time transfusions. In the LV and septal walls, all myocardial velocities were significantly increased compared to normal values, whereas in the RV only Sm was increased before IUT (z-scores 0.26–0.52). In first time IUTs, there was a negative correlation between LV Em (rho = − 0.61, *p* = 0.036) and LV Em/Am (rho = − 0.82, *p* = 0.001) z-scores and hemoglobin before IUT. The peak myocardial velocities that were increased before IUT decreased, whereas LV Em/Am increased significantly after IUT.

**Conclusions:**

This study showed that peak myocardial velocities assessed by cTDI are increased in fetuses before IUT reflecting the physiology of hyperdynamic circulation. In these fetuses, the fetal heart is able to adapt and efficiently handle the volume load caused by IUT by altering its myocardial function.

## Background

Chronic anemia increases the demands on the fetal heart to increase cardiac output and maintain adequate tissue oxygenation. This hyperdynamic cardiovascular adaptation is characterized by cardiomegaly, increased blood flow velocities, decreased vascular resistance and, if left untreated, cardiac dysfunction, hydrops and eventually death [1–4]. To prevent fetal deterioration, anemia is treated with intravascular intrauterine transfusions (IUT) [5, 6]. However, IUT temporarily also subjects the fetal heart to another type of strain when a large, high-viscosity volume is infused during a short period of time.

Current knowledge about cardiac function in anemic fetuses undergoing IUT is mainly derived from assessment of blood flow, whereas surprisingly little is known about the performance of the myocardium. Decreased cardiac output has been demonstrated following IUT when assessing blood flow, but data also indicate that the decrease might be transient [7–9]. Only a few studies have focused on how the myocardium and the pump function of the fetal heart is affected during anemia and IUT. Michel et al. showed an increase in peak systolic myocardial strain in anemic fetuses that partially normalized after IUT [10, 11].

Color tissue Doppler imaging (cTDI) is an echocardiographic technique evaluating myocardial motion that can be used to derive information on variables describing fetal cardiac function such as longitudinal myocardial velocities and cardiac cycle time intervals [12–14]. Despite limitations, cTDI is a promising method for assessing fetal cardiac function. However, its routine clinical application is hampered by a cumbersome and time-consuming off-line analysis that has been partly solved through the use of an automated analysis of myocardial velocities [15–17].

Thus, the aim of this study was to evaluate myocardial function before and after IUT using cTDI with an automated analysis software to identify physiological changes caused by short-term volume load in fetuses under-going transfusion.

## Methods

Pregnant women undergoing IUTs were recruited from the Center for Fetal Medicine at Karolinska University Hospital between 2009 and 2016. Exclusion criteria were multiple pregnancy, major fetal structural/chromosomal abnormality or intraperitoneal transfusion. Gestational age was based on measurement of crown rump length or biparietal diameter in the first trimester or biparietal diameter in the second trimester according to local guidelines. The study was approved by the Stockholm Regional Ethics Committee (DNr 2012/895–31/4 and 2009/1617–31/2) and all women gave their informed consent.

Maternal characteristics, medical history and perinatal outcome were recorded. A transabdominal ultrasound examination was performed for the estimation of fetal weight and measurement of peak systolic velocity in the middle cerebral artery (MCA PSV) using a Voluson E8 ultrasound machine with a 2–5 MHz curved array transducer (GE Healthcare, Zipf, Austria). MCA PSV was recorded and adjusted to multiple of median (MoM) for each gestational age to non-invasively assess the likelihood of fetal anemia as described by Mari et al. [18, 19]. Fetal hydrops was defined as the presence of fluid in at least two body compartments, i.e. ascites, subcutaneous edema, pleural or pericardial effusion.

IUT was performed according to standard techniques and the local protocol for intravascular transfusions has been described previously [20]. Briefly, a 20-gauge needle was inserted into the umbilical vein, either in the intrahepatic portion or at the cord insertion in women with an anterior placenta. Fetal analgesia with alfentanil (0.015 mg/kg) intramuscularly with a 22-gauge needle and fetal paralysis with succinylcholine (5 mg/kg) were applied when transfusion included penetration of the fetal skin with the needle. No antibiotics or tocolysis were given to the mother. Fetal blood sampling was done and packed red blood cells with a hematocrit of 75–85% were transfused intravenously. The volume of blood given was based on fetal weight, estimated fetoplacental blood volume, donor hematocrit and severity of anemia [21, 22]. Before transfusion a complete blood count was obtained by a hematology instrument (PocH-100i, Sysmex Corp.). The hemoglobin value was transformed to multiple of median (Hb MoM). Fetal anemia was defined as mild (0.84–0.65 MoM), moderate (0.65–0.55 MoM) or severe (< 0.55 MoM) [19].

**Fig. 1:**
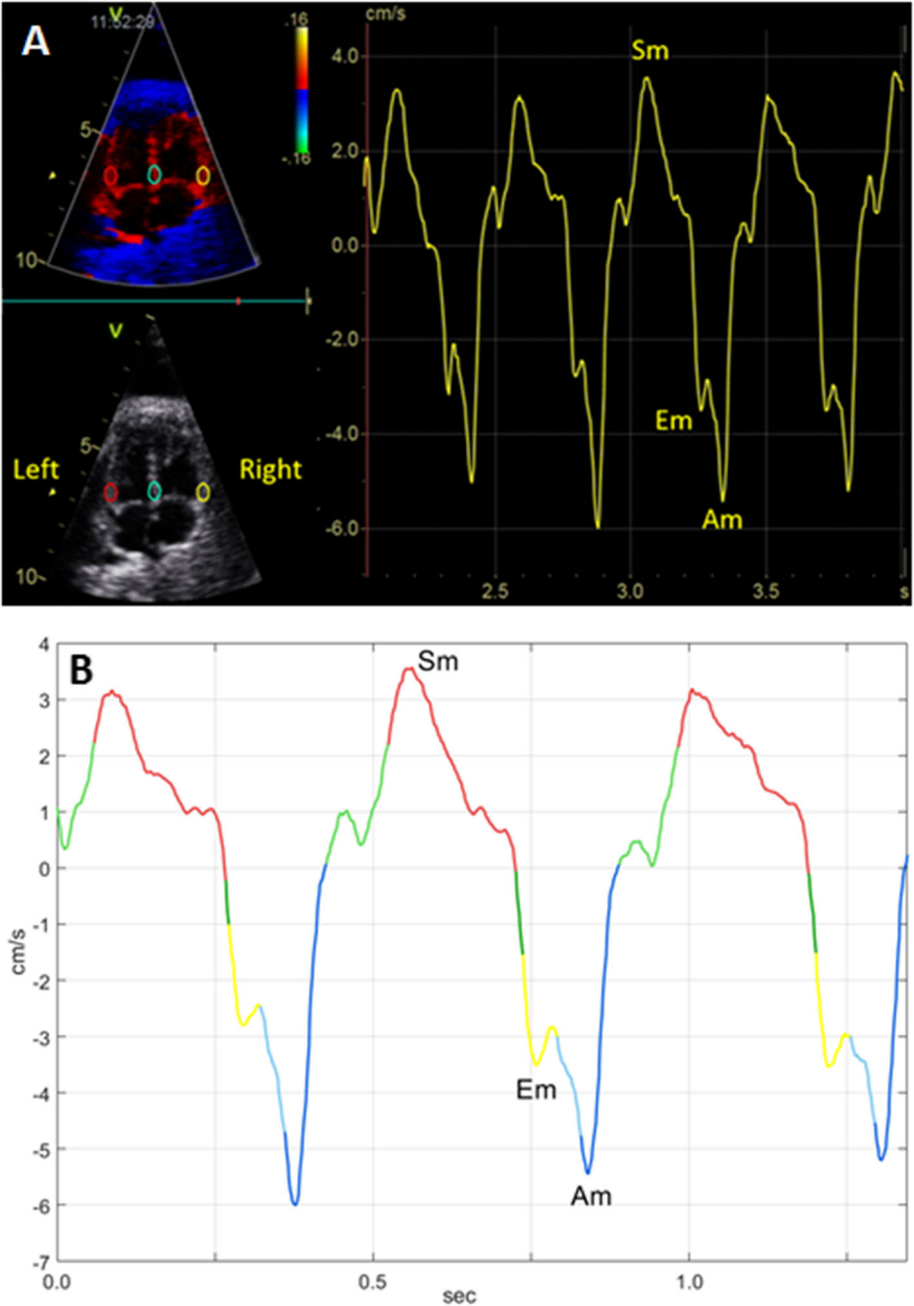
**a**. The position of regions of interest (ROI) with size 6 × 4 mm in an anemic fetus before intrauterine transfusion at 34 weeks of gestation. The left panel indicates the position of ROIs in the four-chamber view of the fetal heart at end-diastole. The right panel shows the myocardial velocity trace derived from the right ventricular wall. **b**. The same myocardial velocity trace analyzed by the automated algorithm. Colors indicate different phases in the cardiac cycle: dark blue – atrial contraction, light green – pre-ejection, red – ventricular ejection, dark green – post-ejection, yellow – rapid ventricular filling and light blue – slow ventricular filling. Sm, peak myocardial velocity during ventricular ejection (systole); Em, peak myocardial velocity during rapid ventricular filling; Am, peak myocardial velocity during atrial contraction

Fetal echocardiography was performed before and within 2.5 h after IUT (median 69 min, interquartile range 54–79) by two experienced operators (KFW and LH) using a GE Vivid-i ultrasound imaging system with a 3S-RS (1.9–3.8 MHz) phased array transducer (GE Vingmed Ultrasound AS, Horten, Norway) or a Vivid S6 ultrasound imaging system with a M4S-RS (1.9–4.1 MHz) phased array transducer (GE CV Ultrasound, Haifa, Israel). All cTDI recordings were obtained in an apical or basal four-chamber view and cine-loops of consecutive cardiac cycles were stored for off-line analysis. Acquisition was optimized to obtain a high frame rate and the incident angle was kept as close to the long-axis of the heart as possible (deviation always <30^o^). Off-line analysis was performed in EchoPAC version 201 (GE Vingmed Ultrasound AS, Horten, Norway). Regions of interest (ROIs) were placed at the atrioventricular plane (AV-plane) in the left (LV) and right ventricular (RV) walls and the interventricular septum (IVS) (Fig. 1). ROI size was adjusted to gestational age [15]. With the aim to reduce variability all ROIs were placed by one operator (LH) and positioned with the AV-plane reaching approximately one third into the ROI at end-systole.

The myocardial velocity traces were subsequently exported from EchoPAC to MATLAB (R2015a, MathWorks, MA, USA), as a text file covering all available velocity data and further analyzed by an automated algorithm also developed into an automated analysis software. This is a fully automated procedure and no manual marking of the traces is needed once the velocity traces are transferred to MATLAB. Moreover, the algorithm compares all identified heart beats and uses that information in identification of cardiac time events. It can also compare different segments, i.e. the IVS, LV and RV walls, over time for more exact definitions [23–25].

The peak myocardial velocity during atrial contraction (Am), ventricular ejection/systole (Sm) and rapid ventricular filling/early diastole (Em) were automatically obtained (Fig. 1). The Em/Am ratio was calculated. Fusion of Em and Am can take place at high heart rates, and such fusion events are detected by the algorithm. When fusion events are detected in a cardiac cycle the Em and Am cannot be separated and the value is omitted. All cardiac cycles available were evaluated by the algorithm and an average from all available cardiac cycles calculated. All measurements were performed for the LV, RV and IVS separately.

### Statistical analysis

Data analysis was performed using IBM SPSS Statistics for Windows, version 23.0 (IBM Corp., Armonk, N.Y., USA) and MATLAB. Continuous variables were presented as mean ± standard deviation (SD) or median (interquartile range) as appropriate. Categorical variables were presented as absolute values and percentage [n (%)].

All cTDI variables were converted to z-scores [(measurement – predicted mean)/ predicted SD] by using published gestational age specific reference data obtained from normal pregnancies by the same technique [17]. Delta (Δ) z-scores were calculated as (z-score after IUT - z-score before IUT).

Mean z-scores and 95% confidence intervals (CI) were presented on measurements before IUT in all IUTs to assess differences between values compared to reference data. Spearman’s correlation (rho) was calculated between the Hb and MCA PSV MoMs before IUT, on one hand, and cTDI z-scores on the other hand. Changes in variables before and after IUT were assessed using Wilcoxon signed rank test for related samples for first and all IUTs. Statistical significance was set to *p* < 0.05.

## Results

**Fig. 2:**
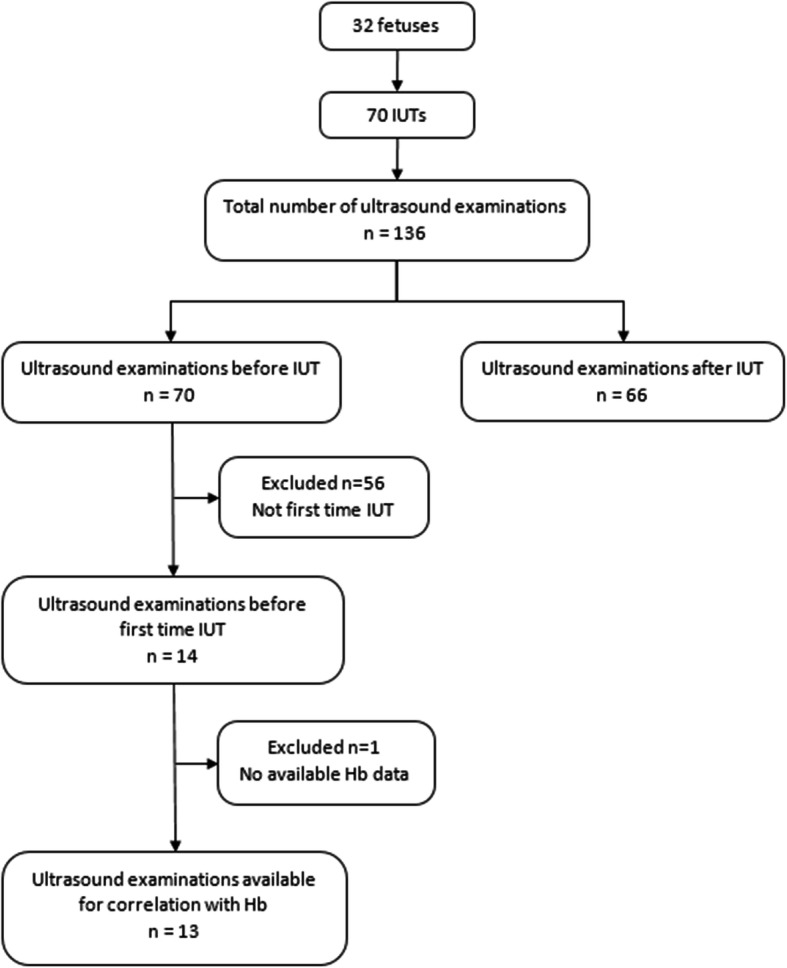
Flow chart describing the ultrasound examinations included in the study. IUT, intrauterine transfusion; Hb, hemoglobin

This study included 32 singleton fetuses that underwent a total of 70 IUTs with a mean of 2.2 ± 1.7 (range, 1–8) transfusions per fetus. cTDI recordings were obtained both before and after IUT in 66, and in four only before IUT. Fourteen of the 70 IUTs were first time transfusions, whereof one was not examined after IUT. In one of these first transfusions there was no hemoglobin value available before IUT. For details see Fig. 2.

Demographic details and clinical characteristics of the study population, transfusions and pregnancy outcome are shown in Table 1. There were 27 fetuses with anemia due to alloimmunization undergoing 63 IUTs. The most common maternal antibody was anti-D (*n* = 22) of which nine in combination with another antibody. Five fetuses underwent seven transfusions due to other causes. One fetus had a suspected Diamond-Blackfan anemia that was postnatally confirmed. Postnatal investigation showed suspicion of Chronic Infantile Neurological and Auricular syndrome in one baby but this was not genetically verified [26]. In three babies the etiology of the anemia remained unknown and persisted postnatally in one case.

**Table 1:** Clinical characteristics of the study population (*n* = 32), intrauterine transfusions (*n* = 70) and pregnancy outcomes

*Variable*	*Value*
Maternal data	
Age (years)	31.2 ± 5.3
BMI (kg/m^2^)	24.1 ± 3.3
Nulliparous	4 (12.5)
Immunization^a^	27 (84.4)
IUT data	
Number of IUTs per fetus	2.2 ± 1.7
Site of tranfusion:	
Intrahepatic	41 (58.6)
Cord insertion in placenta	29 (41.4)
Mean GA at IUT (weeks)	28.6 ± 4.7
Hemoglobin before IUT (g/L)	88.6 ± 28.7
Hemoglobin after IUT (g/L)	141.7 ± 23.9
Degree of anemia: (*n* = 69)	
None	20 (29.0)
Mild	23 (33.3)
Moderate	9 (13.0)
Severe	17 (24.6)
Infused volume (mL)	47.1 ± 23.9
Pregnancy outcome	
GA at delivery (weeks)	35.7 ± 1.4
Pre-term delivery < 37 weeks	28 (87.5)
Mode of delivery:	
Normal vaginal delivery	11 (34.4)
Elective cesarean section	8 (25.0)
Emergency cesarean section	13 (40.6)
Birth weight (g)	2793 ± 443
Female babies	15 (46.9)
Cord arterial pH (*n* = 13)	7.27 ± 0.10
5-min Apgar score < 7	2 (6.3)

Data are given as mean ± SD or *n* (%). BMI, body mass index; IUT, intrauterine transfusion; GA, gestational age

^a^Five fetuses with other causes to anemia

There were 28 preterm deliveries but only two women delivered before week 34 + 0. Fetal hydrops was present at three (4.3%) IUTs in three fetuses. Isolated ascites was present at eight (11.4%) IUTs in six fetuses.

Of the 136 ultrasound examinations performed before or after IUT, the position of the heart was apical in 59 (43%) scans and basal in 77 (57%) scans. The frame rate was 207.7 ± 13.1 frames/sec with a minimum of 173 frames/sec. When extreme outliers in cTDI variables were identified the individual cTDI recordings and velocity traces were visually inspected. Values were omitted if the trace was of insufficient quality for the functioning of the algorithm. This together with missing values due to fusion of Em and Am waves resulted in a mean of 68 observations per variable before IUT (range, 64–70). An average of 9.1 ± 1.7 cardiac cycles were analyzed per myocardial velocity trace.

### Myocardial velocities before IUT compared to reference ranges

**Fig. 3:**
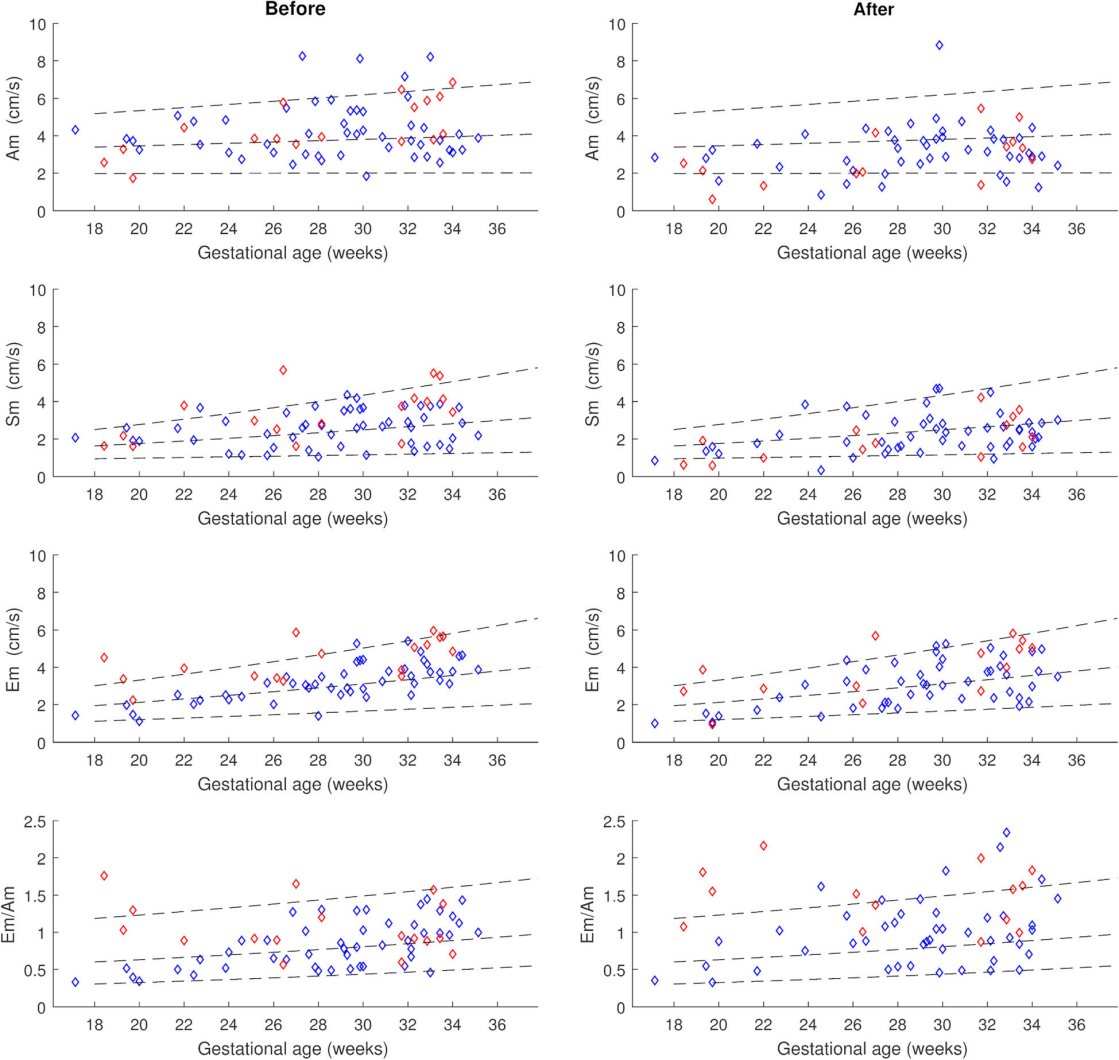
Graphs demonstrating myocardial velocities (absolute values) before and after intrauterine transfusion obtained from color tissue Doppler imaging in the left ventricular wall. Red squares indicate fetuses with severe anemia. Dotted lines represent reference ranges with 5th, mean and 95th centiles. Am, peak myocardial velocity during atrial contraction; Sm, peak myocardial velocity during ventricular ejection (systole); Em, peak myocardial velocity during rapid ventricular filling

Myocardial velocities before all 70 IUTs are presented in Table 2. Am, Sm and Em were all significantly increased in the LV and IVS, whereas in the RV only Sm reached statistical significance compared to our reference standard. Details for myocardial velocities in the LV wall are illustrated in Fig. 3.

**Table 2:** Myocardial velocities before intrauterine transfusion (*n* = 70). Absolute values and z-scores

*Variable*	*LV*		*IVS*		*RV*	
	*Mean (*±SD)	*Mean z-score (95% CI)*	*Mean (*±SD)	*Mean z-score (95% CI)*	*Mean (*±SD)	*Mean z-score (95% CI)*
	(cm/sec)		(cm/sec)		(cm/sec)	
Myocardial velocities						
Am	4.27 (±1.46)	0.31^a^ (0.06–0.56)	4.03 (±1.01)	0.28^a^ (0.08–0.48)	5.83 (±1.46)	0.12 (−0.16–0.40)
Sm	2.77 (±1.11)	0.34^a^ (0.08–0.61)	2.84 (±0.68)	0.52^a^ (0.28–0.77)	3.66 (±1.14)	0.30^a^ (0.03–0.57)
Em	3.55 (±1.19)	0.48^a^ (0.24–0.73)	3.38 (±0.96)	0.26^a^ (0.05–0.47)	4.20 (±1.28)	0.12 (−0.12–0.35)
Em/Am ratio	0.90 (±0.36)	0.20 (−0.05–0.44)	0.87 (±0.28)	−0.01 (− 0.21–0.18)	0.76 (±0.26)	0.09 (− 0.15–0.34)

Values are presented as absolute values and z-scores created from published reference data

^a^indicates significance according to 95% confidence interval (CI)

LV, left ventricular wall; IVS, interventricular septum; RV, right ventricular wall; Am, peak myocardial velocity during atrial contraction; Sm, peak myocardial velocity during ventricular ejection; Em, peak myocardial velocity during rapid ventricular filling; SD, standard deviation

### Correlation between myocardial velocities and Hb and MCA PSV before IUT

**Fig. 4:**
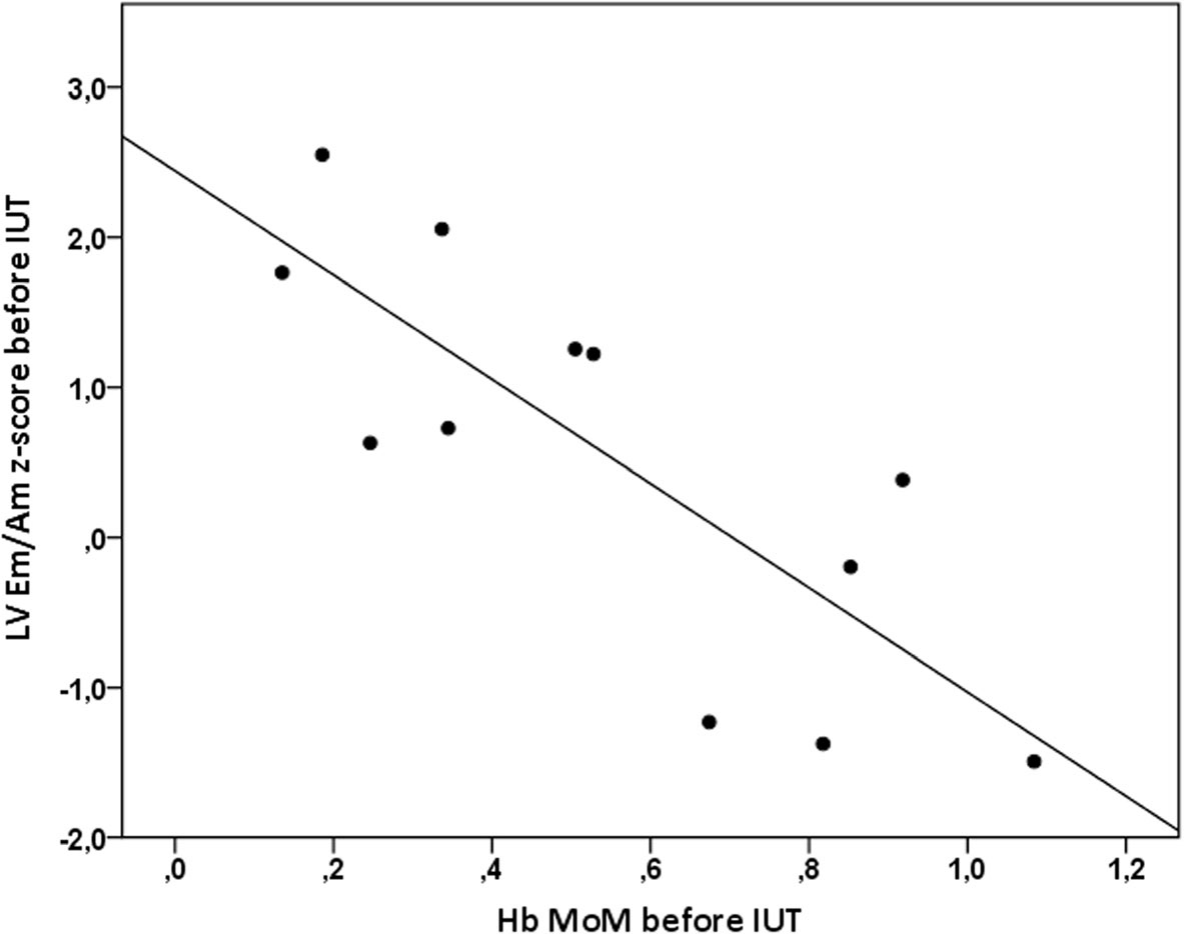
Correlation between left ventricular (LV) Em/Am z-scores and hemoglobin value expressed as multiple of median (Hb MoM) before first intrauterine transfusions (IUT)

When analyzing observations made before first IUTs in the 12 cases with available Hb values and cTDI measurements, we observed a negative correlation between Hb and LV Em (rho = − 0.61, *p* = 0.036) as well as LV Em/Am (rho = − 0.82, *p* = 0.001, Fig. 4). When analyzing all IUTs, weaker negative correlations were found between Hb and Sm, Em, Em/Am in LV and Sm in IVS (rho = − 0.25 - -0.49, *p* < 0.05).

**Fig. 5:**
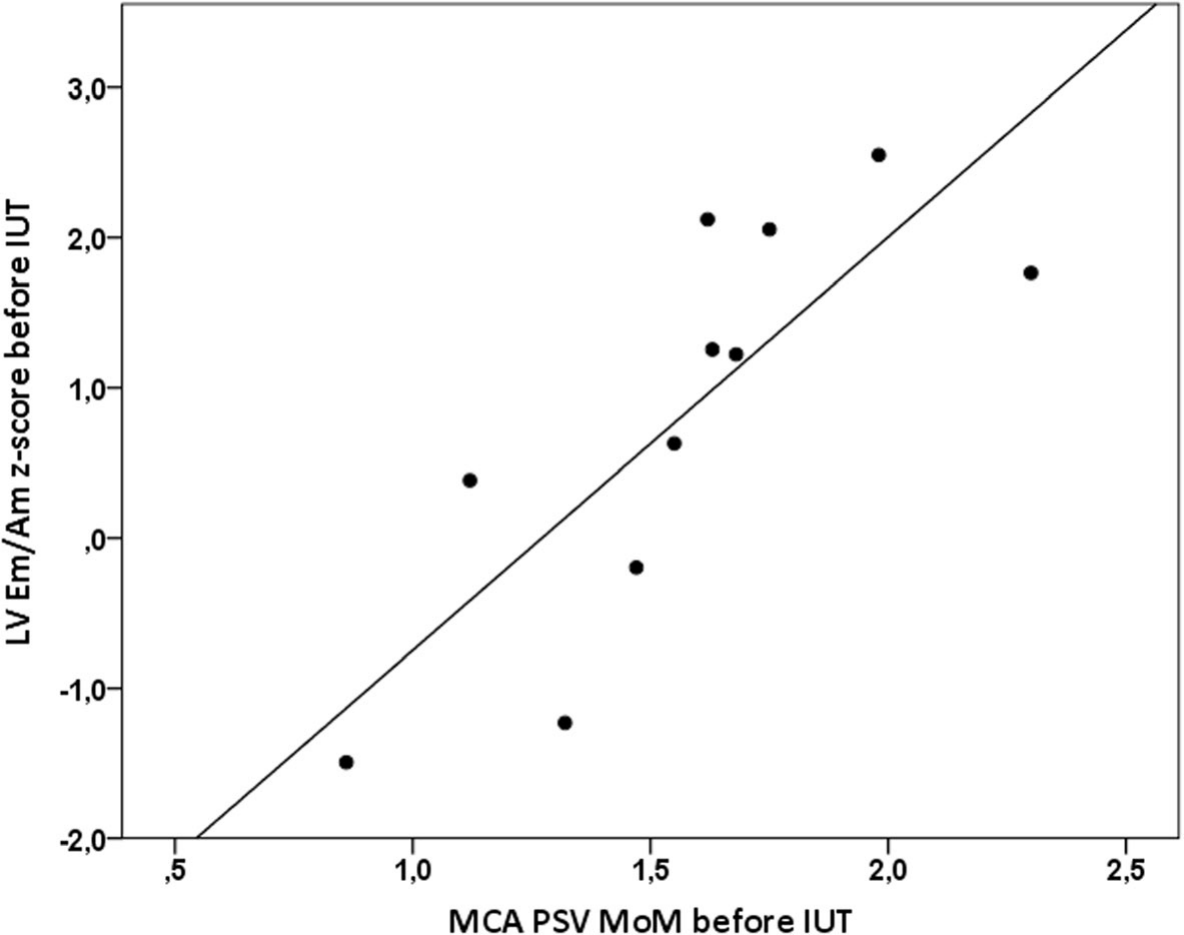
Correlation between left ventricular (LV) Em/Am z-score and peak systolic velocity in the middle cerebral artery expressed as multiple of median (MCA PSV MoM) before first intrauterine transfusions (IUT)

Correlation analysis between z-scores of cTDI variables and MCA PSV before first IUTs showed a strong positive correlation between MCA PSV and LV Em (rho = 0.66, *p* = 0.03) as well as LV Em/Am (rho = 0.84, p = 0.001, Fig. 5). When analyzing all IUTs, weaker positive correlations were found with LV Em and Em/Am (rho = 0.34, 0.39, p < 0.05).

### Comparison of myocardial velocities before and after IUT

Fetuses receiving their first IUTs showed a significant decrease in Am and Sm in the LV as well as Sm and Em in IVS following IUT with median Δ z-scores − 0.69- -1.50; *p* < 0.05.

In the group of fetuses receiving their first time transfusion that had Hb values as well as pre- and post-transfusion cTDI measurements recorded (*n* = 12), the median Hb MoM was 0.60 (range, 0.14–1.08). Three fetuses had mild anemia, six fetuses had severe anemia and three fetuses had no anemia. In the sub-group of anemic fetuses (*n* = 9) the only variables that showed a significant change after transfusion was Sm which decreased from 1.84 cm/s to 1.53 cm/s in the LV (*p* = 0.05) and 2.50 cm/s to 1.74 cm/s in the IVS (*p* = 0.017).

When assessing all IUTs with available data before and after IUT, we found a significant decrease in heart rate from 140.4 ± 9.1 bpm before to 132.7 ± 14.4 bpm (*p* = 0.001) following transfusion. All peak myocardial velocities except Am and Em in the RV decreased significantly after IUT (median Δ z-scores − 0.34- -0.97; *p* < 0.01). The LV Em/Am ratio increased after IUT (median Δ z-score + 0.58; *p* < 0.001) whereas the other ratios did not change. Table 3. Details for the LV wall are also illustrated in Fig. 3.

**Table 3:** Difference in myocardial velocities before and after intrauterine transfusion (*n* = 66). Absolute values and z-scores

*Variable*	*LV*		*IVS*		*RV*	
	*Median* ^b^ *(*IQ)	*Median* ^b^ *z-score* *(*IQ)	*Median* ^b^ *(*IQ)	*Median* ^b^ *z-score* *(*IQ)	*Median* ^b^ *(*IQ)	*Median* ^b^ *z-score* *(*IQ)
	(cm/sec)		(cm/sec)		(cm/sec)	
Myocardial velocities						
Am	−1.03 (1.52)	−0.75^a^ (1.54)	− 0.62 (1.41)	− 0.61^a^ (1.29)	−0.47 (2.36)	− 0.40 (1.73)
Sm	−0.52 (1.13)	− 0.62^a^ (1.30)	−0.49 (0.64)	− 0.97^a^ (1.18)	−0.45 (1.23)	− 0.49^a^ (1.45)
Em	−0.41 (1.08)	− 0.38^a^ (1.12)	−0.34 (1.14)	− 0.34^a^ (1.16)	−0.11 (2.00)	− 0.13 (1.61)
Em/Am ratio	+ 0.23 (0.55)	+ 0.58^a^ (1.43)	+ 0.05 (0.50)	+ 0.19 (1.55)	+ 0.002 (0.32)	+ 0.01 (1.35)

Values are presented as absolute values and z-scores created from published reference data

^a^indicates significance according to Wilcoxon signed rank test for related samples *p* < 0.01

LV, left ventricular wall; IVS, interventricular septum; RV, right ventricular wall

^b^, difference between measurement after minus before IUT; IQ; interquartile range

Am, peak myocardial velocity during atrial contraction; Sm, peak myocardial velocity during ventricular ejection; Em, peak myocardial velocity during rapid ventricular filling

## Discussion

This study showed that all peak myocardial velocities in the LV and IVS and Sm in the RV wall are increased before IUT compared to normal values, reflecting the hyperdynamic circulation. The same myocardial velocities, as well as the heart rate, also decreased significantly after IUT. We also demonstrated a negative correlation between Hb concentration and LV Em and LV Em/Am before transfusion in all IUTs and more prominently when only first time IUTs were assessed. This study also showed that the effect of IUT on the fetal myocardium can be assessed using cTDI with automated analysis.

The increase in myocardial velocities before IUT, is in accordance with the hyperdynamic circulation associated with anemia that has been described by other investigators recording fetal blood flow velocities [2, 27, 28]. Interestingly, myocardial velocity during rapid ventricular filling (Em) seemed increasingly enhanced compared to the myocardial velocity during atrial contraction (Am) as the degree of anemia increased, suggesting an enhanced ability for relaxation. This resulted in a strong negative correlation (rho = − 0.82) between Hb and LV Em/Am ratio before first transfusions. Blood flow velocities in the MCA, the thoracic aorta and across the mitral and aortic valves have previously been demonstrated to correlate with hemoglobin levels in anemic fetuses [2, 19, 29]. In accordance with our present observations, Rizzo et al. have also shown a trend towards increased E/A ratios compared to normal controls using the measurement of blood flow velocities across the atrioventricular valves before IUT in a small group of previously transfused fetuses [8].

We observed a decrease of systolic and diastolic myocardial velocities and a significant increase in LV Em/Am ratio after IUT in accordance with the findings reported using fetal cardiac blood flow velocity variables [8]. A decrease in cardiac output immediately after IUT has previously been described by other investigators [7, 8, 30]. The most obvious explanation to the decrease in several of the peak myocardial velocities after IUT is the reversal of the hyperkinetic circulation as hemoglobin level and oxygen carrying capacity increase. However, the reduction of myocardial velocities was greater than expected suggesting other effects on cardiac function. This might be due to the fast volume expansion with packed red blood cells that might also have an immediate effect on afterload [31] known to be poorly tolerated by the fetal heart, and result in a decreased cardiac output [32, 33] and, thus, possibly further diminishing myocardial velocities.

The strength of this study is that it describes fetal cardiac physiology in fetuses before and after IUT with a focus on myocardial function that has not been well studied. It also demonstrates the potential of automation in facilitating the use of cTDI in the evaluation of fetal myocardial function that can be applied as a tool for the assessment of fetal cardiac physiology under different conditions.

Our study does have some limitations. Multiple measurements were performed in the same fetus when evaluating all IUTs, which may introduce some bias when assessing the correlation between myocardial velocities and degree of fetal anemia. Therefore, we evaluated first IUTs separately even though the numbers were few. The study group was also heterogeneous with varied etiologies and varying degrees of anemia that might influence the results. It is also important to acknowledge that evaluation of cardiac function has been performed close after, and with varying intervals from, the IUT and that several investigators did not demonstrate changes in cardiac variables when longer time had elapsed [1, 9, 34]. Moreover, cTDI has limitations, such as angle dependency influencing velocity measurements and subjectivity in ROI placements, although the latter might be solved by new automation techniques, such as artificial intelligence technology.

However, the current study demonstrated that the rapid physiological adaptation at fetal transfusions well can be monitored by the automated method we utilize in the present study. It remains to be established how this promising method will perform in more chronic disorders such as intrauterine growth restriction. Preliminary studies suggest that the method is sensitive enough to evaluate physiological adaptation at this condition.

## Conclusions

This study showed that peak myocardial velocities assessed by cTDI are increased in fetuses before IUT reflecting the physiology of hyperdynamic circulation. It also showed that the fetal heart is able to adapt and efficiently handle the volume load caused by IUT by altering its myocardial function.

With advances in automation, cTDI could be more readily applicable to study changes in fetal cardiac physiology.

## Data Availability

The datasets generated and/or analyzed during the current study are not publicly available due the small and specific number of cases that might be traceable to patients.

## Abbreviations

IUT: Intrauterine transfusion
cTDI: Color tissue Doppler imaging
MCA PSV: Middle cerebral artery peak systolic velocity
MoM: Multiple of median
Hb: Hemoglobin
ROI: Region of interest
AV-plane: Atrioventricular plane
LV: Left ventricular/left ventricle
RV: Right ventricular/right ventricle
IVS: Interventricular septum
Am: Peak myocardial velocity during atrial contraction
Sm: Peak myocardial velocity during ventricular ejection
Em: Peak myocardial velocity during rapid ventricular filling
SD: Standard deviation
CI: Confidence interval
BMI: Body mass index
GA: Gestational age
Bpm: Beats per minute
